# The status of intercellular junctions in established lens epithelial cell lines

**Published:** 2012-12-12

**Authors:** Alpana Dave, Jamie E. Craig, Shiwani Sharma

**Affiliations:** Department of Ophthalmology, Flinders University, Bedford Park, SA 5042, Australia

## Abstract

**Purpose:**

Cataract is the major cause of vision-related disability worldwide. Mutations in the crystallin genes are the most common known cause of inherited congenital cataract. Mutations in the genes associated with intercellular contacts, such as Nance-Horan Syndrome (*NHS*) and Ephrin type A receptor-2 *(EPHA2*), are other recognized causes of congenital cataract. The *EPHA2* gene has been also associated with age-related cataract, suggesting that intercellular junctions are important in not only lens development, but also in maintaining lens transparency. The purpose of this study was to analyze the expression and localization of the key cell junction and cytoskeletal proteins, and of NHS and EPHA2, in established lens epithelial cell lines to determine their suitability as model epithelial systems for the functional investigation of genes involved in intercellular contacts and implicated in cataract.

**Methods:**

The expression and subcellular localization of occludin and zona occludens protein-1 (ZO-1), which are associated with tight junctions; E-cadherin, which is associated with adherence junctions; and the cytoskeletal actin were analyzed in monolayers of a human lens epithelial cell line (SRA 01/04) and a mouse lens epithelial cell line (αTN4). In addition, the expression and subcellular localization of the NHS and EPHA2 proteins were analyzed in these cell lines. Protein or mRNA expression was respectively determined by western blotting or reverse transcription-polymerase chain reaction (RT–PCR), and localization was determined by immunofluorescence labeling.

**Results:**

Human SRA 01/04 and mouse αTN4 lens epithelial cells expressed either the proteins of interest or their encoding mRNA. Occludin, ZO-1, and NHS proteins localized to the cellular periphery, whereas E-cadherin, actin, and EPHA2 localized in the cytoplasm in these cell lines.

**Conclusions:**

The human SRA 01/04 and mouse αTN4 lens epithelial cells express the key junctional proteins. The localization patterns of these proteins suggest that these cell lines form tight junctions but do not form E-cadherin-based adherence junctions. These data further indicate that the regulatory role of NHS in actin remodeling, suggested in another study, is cell type dependent. In conclusion, the SRA 01/04 and αTN4 lens epithelial cell lines model some characteristics of an epithelium.

## Introduction

Cataract remains the leading cause of blindness worldwide. Mutations in 29 different genes with diverse functions are known to cause inherited congenital cataract, the rare form of cataract, with mutations in the crystallin genes being the major reported cause [1-3].

More recently, mutations in genes involved in intercellular adhesion have emerged as another cause of inherited congenital cataract, indicating the importance of intercellular contacts in lens development and cataractogenesis. The full-length isoform of the Nance-Horan Syndrome (*NHS*) gene, NHS-A, associates with epithelial tight junctions; we have reported that mutations in this gene cause X-linked syndromic cataract in NHS [4-6]. The protein encoded by the Ephrin type A receptor-2 (*EPHA2*) gene regulates epithelial cell adhesion [7]. We and other groups found that mutations in this gene cause autosomal dominant and recessive forms of congenital cataract [8-10]. The *EPHA2* gene is also genetically associated with age-related cortical cataract [9,11,12]. Both, the NHS and EPHA2 proteins are expressed in the mammalian lens [4,11]. The NHS-A protein localizes at cell-cell contacts in the anterior lens epithelium, whereas EPHA2 localizes to cell-cell contacts in the equatorial lens epithelium and cortical fiber cells [5,11,13]. The mechanisms of cataractogenesis involving the *NHS* and *EPHA2* genes are poorly understood. Preliminary investigations of the function of NHS and mechanism of cataract formation in NHS were undertaken in nonocular ex vivo models of mammalian epithelium, such as Madin-Darby canine kidney (MDCK), and human colorectal adenocarcinoma (Caco-2) cells [14,15]. This is primarily because whether the lens epithelial cell lines derived from the mammalian lens, model an epithelium ex vivo is unknown. Hence, the aim of this study was to determine expression and localization of key cell-cell contact molecules in established lens epithelial cell lines and ascertain whether these cell lines model the mammalian epithelium ex vivo.

Epithelial cells adhere together through three types of junctional complexes—tight junctions (TJs), adherence junctions (AJs), and desmosomes—that are respectively distributed apically to basally along the lateral cell membrane [16]. TJs define epithelial cell polarity, regulate paracellular permeability, and confer barrier function to the epithelia [17]. AJs are responsible for cell-cell adhesion and maintaining the integrity of TJs [18]. Desmosomes provide anchoring sites for cytoskeletal structures, the intermediate filaments [19]. TJs are composed of transmembrane proteins, occludins, claudins, and junctional adhesion molecules that interact with the cytoplasmic plaque proteins such as zonula occludens (ZO) adaptor proteins, and with signaling proteins that regulate cell proliferation, migration, and differentiation [20]. TJs are linked with the actin cytoskeleton via the ZO proteins. AJs are composed of cadherin- and nectin-based adhesion complexes [18]. They are also linked with the actin cytoskeleton.

In the present study, we investigated the expression and localization of the TJ proteins occludin and ZO-1, the AJ protein E-cadherin, and the cytoskeletal protein β-actin in two lens epithelial cell lines, namely human SRA 01/04 [21] and mouse αTN4 [22] cells. The investigation also included the NHS and EPHA2 proteins. The SRA 01/04 cells have been immortalized by Simian virus 40 (SV40) large T antigen transformation [21] and αTN4 cells have been derived from the lens epithelium of transgenic mice expressing SV40 large-T antigen transgene under the crystallin αA promoter [22]. The presented data suggest that the two lens epithelial cell lines retain some characteristics of an epithelium in culture.

## Methods

### Mammalian cell culture

The human SRA 01/04 and mouse αTN4 lens epithelial cells were kind gifts from Dr. Venkat Reddy (Kellogg Eye Institute, University of Michigan, MI) and Dr. Paul Russell (University of California, Davis, CA), respectively. Both of the cell lines were cultured in Dulbecco’s Modified Eagle’s medium (DMEM; GIBCO, Life Technologies Australia Pty Ltd., Mulgrave, Australia) supplemented with 10% fetal bovine serum and penicillin/streptomycin. Cell cultures were maintained in a humidified atmosphere at 37 °C and 5% CO_2_.

### Western blot analysis

Cells (4×10^5^ SRA 01/04 or αTN4, per well) were seeded in six-well tissue culture plates. After approximately 48 h, the cells were harvested and cellular proteins extracted using radio-immunoprecipitation assay (RIPA) buffer (10 mM HEPES pH 7.5, 150 mM sodium chloride, 2 mM EDTA, 1% Triton X-100, 0.5% sodium deoxycholate, 0.1% sodium dodecyl sulfate [SDS], 25× protease inhibitor cocktail [Roche Diagnostics Australia Pty Ltd., Castle Hill, Australia], 57 µM phenylmethylsulfonyl fluoride, 2 mM sodium orthovanadate, 10 mM sodium pyrophosphate and 20 mM sodium fluoride). Forty micrograms of total soluble proteins of each cell line were separated on a 10% polyacrylamide gel by SDS–PAGE (PAGE) and transferred onto Hybond C-extra (GE Healthcare Australia and New Zealand, Sydney, Australia). The blot was probed with the mouse monoclonal anti-ZO-1 (1:250; Zymed Laboratories, Life Technologies Australia Pty Ltd., Mulgrave, Australia), anti-occludin (1:250; Zymed Laboratories), anti-E-cadherin (1:2,500; BD Transduction Laboratories, North Ryde, Australia), anti-β-actin (1:5,000, Sigma-Aldrich Pty Ltd., Castle Hill, Australia), or anti-Eck/EphA2 clone D7 (1:500; Upstate Biotechnology, Millipore Australia Pty Ltd., Kilsyth, Australia) primary antibody. Primary antibody binding was detected by hybridization with the sheep anti-mouse IgG–horseradish peroxidase–conjugated (1:1,000; Chemicon Australia Pty Ltd., Boronia, Australia) or donkey anti-mouse IgG–horseradish peroxidase–conjugated (1:1,000; Jackson ImmunoResearch Laboratories Inc., Brisbane, Australia) secondary antibody. MCF-7 breast cancer cell lysate was included as a positive control on western blots probed with the anti-occludin, anti-E-cadherin, and anti-β-actin antibodies. Antibody binding was detected using the enhanced chemulimunescence (ECL) western blotting system (GE Healthcare Australia and New Zealand, Sydney, Australia) or SuperSignal West Pico Chemiluminescent Substrate (Thermo Fisher Scientific, Scoresby, Australia).

### Reverse transcription-polymerase chain reaction

Total RNA from frozen pellets of SRA 01/04 and αTN4 cells was extracted using the RNeasy mini kit (QIAGEN Pty Ltd., Doncaster, Australia) following the manufacturer’s protocol. First strand cDNA synthesis was performed with Superscript III reverse transcriptase (Invitrogen, Life Technologies Australia Pty Ltd., Mulgrave, Australia) as per the manufacturer’s instructions using random hexamers. On the resulting cDNA template, PCR was performed with mouse *Occludin*-specific primers 5′-AAG AGT ACA TGG CTG CTG CTG ATG-3′ (forward) and 5′ CTT AAT TGG AGT GTT CAG CCC AGT-3′ (reverse), and human and mouse *E-cadherin*-specific primers 5′-CTG GGC TGG ACC GAG AGA TT-3′ (forward) and 5′-GCA GTG TAG GAT GTG ATT TCC TG-3′ (reverse) using HotStar Taq (QIAGEN). PCR included denaturation at 95 °C for 15 min and 40 cycles of denaturation at 95 °C for 30 s, annealing at 60 °C for 30 s, and elongation at 72 °C for 30 s, for the amplification of *Occludin* cDNA, as well as denaturation at 95 °C for 15 min and 50 cycles of denaturation at 95 °C for 30 s, annealing at 56 °C for 30 s, and elongation at 72 °C for 30 s for the amplification of *E-cadherin* cDNA.

### Immunofluorescent labeling

Cells (3×10^5^ SRA 01/04 or αTN4) were seeded onto glass coverslips placed in six-well tissue culture plates. Four days later, for immunolabeling, the cells were fixed in 4% paraformaldehyde/ phosphate buffered saline (PBS), permeabilized with 0.4% Triton X-100, blocked with 5% donkey serum, and hybridized with rabbit polyclonal anti-ZO-1 (1:250; Zymed Laboratories), mouse anti-E-cadherin (1:500), rabbit anti-NHS (1:100–200) [5], or mouse monoclonal anti-EphA2 (1:100–500) primary antibody. For the labeling of occludin, the cells were fixed and permeabilized in chilled ethanol/acetone, blocked with PTB (0.1% Triton X-100 and 5% BSA in PBS), and hybridized with rabbit polyclonal anti-occludin antibody (1:250; Zymed Laboratories). Anti-ZO-1, anti-NHS, and anti-occludin antibody binding was detected with Alexa Fluor 488-conjugated anti-rabbit IgG (1:1,000; Molecular Probes, Life Technologies Australia Pty Ltd., Mulgrave, Australia), and binding of anti-EphA2 and anti-E-cadherin antibodies with Alexa Fluor 488-conjugated anti-mouse IgG (1:1,000; Molecular Probes) secondary antibody. Nuclei in E-cadherin-labeled cells were stained with propidium iodide (Molecular Probes) after treatment with RNase A/T1 cocktail (Ambion, Life Technologies Australia Pty Ltd., Mulgrave, Australia). Labeled cells were mounted on microscope slides in buffered glycerol (pH 8.6). Occludin, ZO-1, E-cadherin, and NHS labeling was visualized on an Olympus BX50 epifluorescence microscope using a blue filter and images taken using the AnalySIS software (Olympus, Adelaide, SA, Australia). Propidium iodide staining was visualized using a green filter. EPHA2 labeling was imaged on a Leica TCS SP5 confocal microscope using the LAS Image analysis software (Leica Microsystems Pty Ltd, North Ryde, NSW, Australia). Alexa Fluor 488 was excited with an Argon laser with emission spectrum set at 492–561 nm.

For localization of actin, confluent SRA 01/04 and αTN4 cells grown on glass coverslips were fixed in 4% paraformaldehyde/PBS, permeabilized with 0.4% Triton X-100, stained with phalloidin-tetramethyl rhodamine isothiocyanate (TRITC; 1:250, Sigma-Aldrich Pty Ltd.) for 30 min, washed several times with PBS, and mounted in Prolong Gold Antifade reagent with 4’,6-diamino-2-phenylindole (DAPI; Molecular Probes). Actin staining was imaged by confocal microscopy as explained above. TRITC was excited with DPSS 561 laser line with emission spectrum set at 565–645 nm.

## Results

### Expression analysis

**Figure 1:**
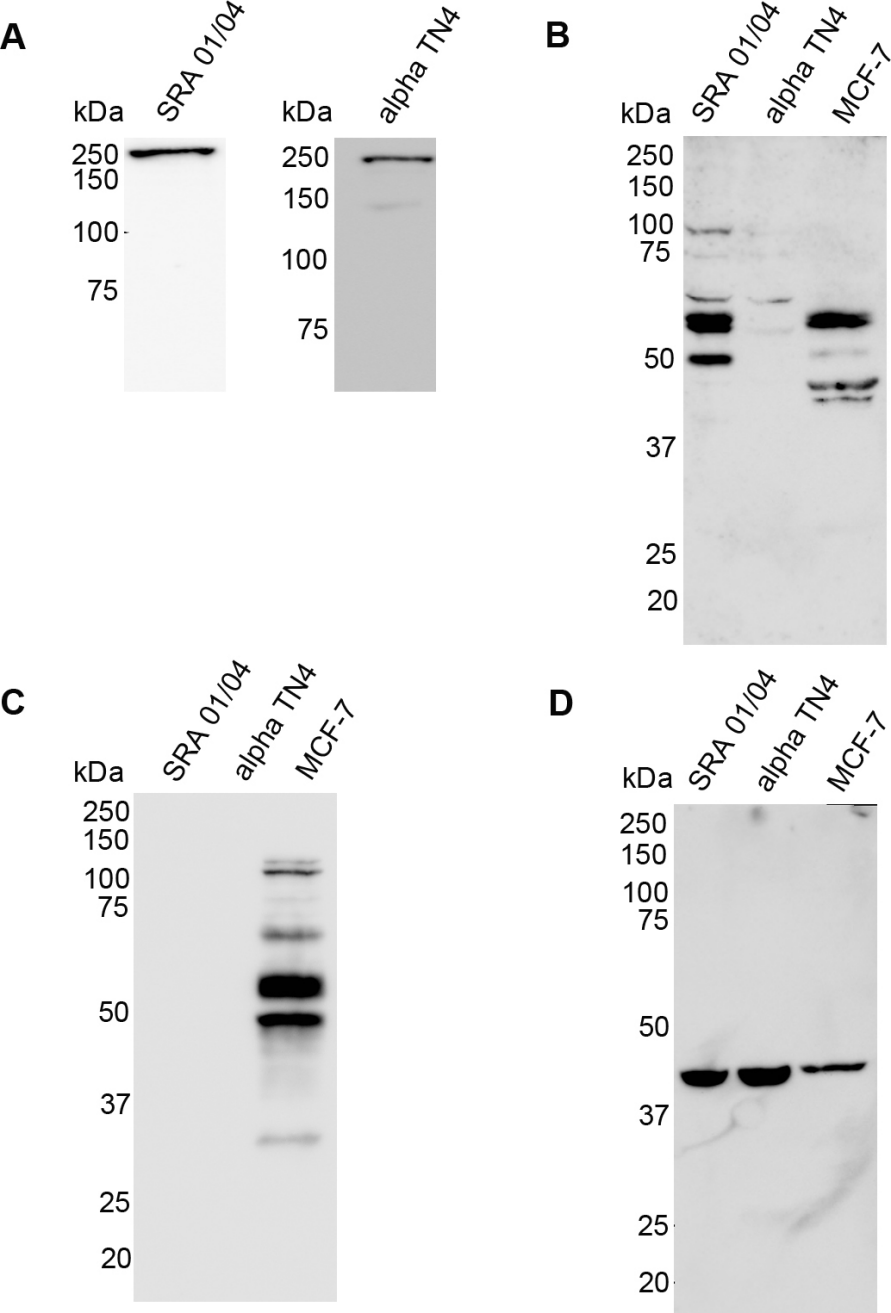
Expression of cellular junctions associated and cytoskeletal proteins in human SRA 01/04 and mouse αTN4 lens epithelial cells. Western blotting was performed on protein lysates of SRA 01/04 and αTN4 cells using the (**A**) anti-zona occludin-1 (ZO-1), (**B**) anti-occludin, (**C**) anti-E-cadherin, and (**D**) anti-β-actin antibodies. Protein lysate of MCF-7 human breast cancer cells was used as a positive control in **B**, **C**, and **D**. **A**: A band of ~220 kilo Daltons (kDa) of the expected size of ZO-1 was detected in each cell line. **B**: A ~60 kDa band corresponding to the expected size of occludin was detected in SRA 01/04 and the positive control MCF-7 cells. A very faint band of similar size was observed in αTN4 cells. The <60 kDa bands in SRA 01/04 and MCF-7 cells likely represent occludin isoforms and >60 kDa bands in SRA 01/04 cells likely represent nondenatured protein. **C**: A ~100 kDa band corresponding to the expected size of the full-length E-cadherin was detected in the positive control MCF-7 cells, but no signal was detected in SRA 01/04 or αTN4 cells. The <100 kDa bands in MCF-7 cells are likely E-cadherin cleavage products. **D**: A ~42 kDa band of the expected size of β-actin was detected in all the cell lines. Molecular masses of protein standards are indicated in kDa.

Expression of the junctional proteins occludin, ZO-1, and E-cadherin, cytoskeletal protein β-actin, NHS and EPHA2 proteins, or their encoding genes in SRA 01/04 and αTN4 cells was respectively analyzed by western blotting or reverse transcription-polymerase chain reaction (RT–PCR). Western blotting of protein lysates of the two cell lines with the anti-ZO-1 antibody revealed a specific protein band of ~220 kDa in each cell line that corresponds with the expected size of the ZO-1 protein (Figure 1A). Hybridization of protein lysates from the two cell lines with the anti-occludin antibody detected a prominent band of ~60 kDa, the expected size of occludin, in SRA 01/04 cells (Figure 1B). A similar sized band was also detected in MCF-7 human breast cancer cells that were used as a positive control (Figure 1B). However, in αTN4 cells, only a faint band of the expected size was detected with this antibody. The bands smaller than 60 kDa in SRA 01/04 and MCF-7 cell lysates likely represent occludin isoforms; those larger than 60 kDa in SRA 01/04 cells may represent incompletely denatured protein. Similar hybridization with the anti-E-cadherin antibody did not reveal any signal in SRA 01/04 and αTN4 cells, but detected a ~100 kDa band corresponding to the expected size of the full-length E-cadherin protein in MCF-7 cells (Figure 1C). E-cadherin is known to undergo proteolytic cleavage [23]; therefore, the protein bands smaller than 100 kDa seen in MCF-7 cells may represent its cleavage products. Hybridization with the anti-β-actin antibody revealed a protein band of ~42 kDa in SRA 01/04 and αTN4 cells, and in the positive control MCF-7 cells (Figure 1D). The detected band corresponds with the expected size of the β-actin protein. The absence of a signal with the anti-E-cadherin antibody in SRA 01/04 and αTN4 cell lysates, indicating that E-cadherin is either not expressed or is expressed at undetectable levels in these cells.

**Figure 2:**
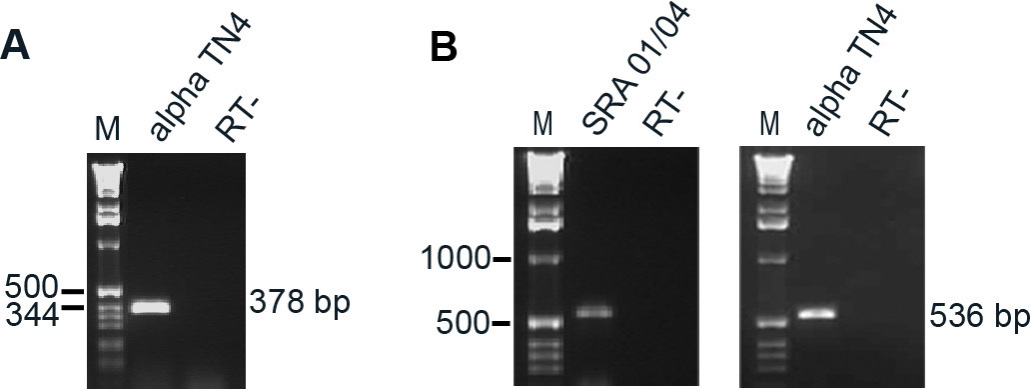
Expression of *Occludin* and *E-cadherin* mRNA in human SRA 01/04 and/or mouse αTN4 lens epithelial cells. mRNA expression was analyzed by reverse transcription-polymerase chain reaction (RT–PCR) using gene specific primers. **A**: *Occludin* expression in αTN4 cells demonstrated by amplification of a PCR product of the expected size of 378 base pairs (bp). **B**: Expression of *E-cadherin* in SRA 01/04 and αTN4 cells demonstrated by amplification of the expected PCR products, respectively, of 536 and 537 bp. The specificity of each PCR product was confirmed by sequencing. Molecular sizes of DNA markers in bp are indicated on the left. M indicates DNA marker and RT- indicates without reverse transcriptase control.

Further, to confirm expression of *Occludin* in αTN4 cells and to determine *E-cadherin* mRNA (mRNA) expression in the two cell lines, RT–PCR was performed using gene specific primers. RT–PCR of mRNA from αTN4 cells with mouse *Occludin*–specific primers revealed, as expected, a product of 378 bp (Figure 2A). Similarly, RT–PCR of mRNA from the two cell lines using the human and mouse *E-cadherin* specific primers, as expected, resulted in products of 536 bp and 537 bp, respectively, in SRA 01/04 and αTN4 cells (Figure 2B). The specificity of each amplified product was confirmed by sequencing. In summary, RT–PCR data confirmed *Occludin* expression in αTN4 cells and demonstrated expression of *E-cadherin* in SRA 01/04 and αTN4 cells.

**Figure 3:**
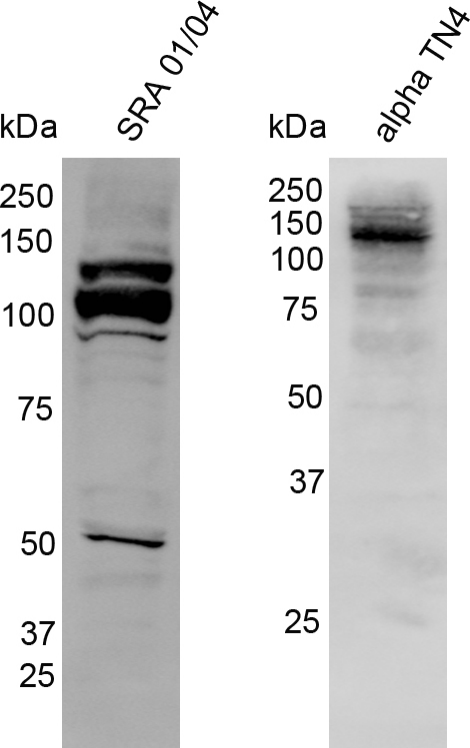
Expression of the EPHA2 protein in human SRA 01/04 and mouse αTN4 lens epithelial cells. Western blotting was performed on cell lysates of each cell line with the anti-EphA2 antibody. The specific band of ~140 kilo Daltons (kDa) in the two cell lines corresponds to the posttranslationally modified form of the protein. The ~108 kDa band in SRA 01/04 cells corresponds to the unmodified form of EPHA2 and the ~50 kDa band is likely a degradation product. The molecular masses of protein standards are indicated in kDa.

Western blotting of cell lysates from SRA 01/04 and αTN4 cells with the anti-EphA2 antibody revealed two bands of ~140 kDa and ~108 kDa in the former, and a band of ~140 kDa in the latter cells (Figure 3). The EPHA2 protein is phosphorylated at several residues and undergoes glycosylation and ubiquitination [24-27]. The ~140 kDa band in both the cell lines likely represents posttranslationally modified form of the protein [7]. The smaller ~108 kDa band observed in SRA 01/04 cells may represent the unmodified form of human EPHA2. The ~50 kDa band in SRA 01/04 cells may be a degradation product.

Together, these data demonstrate that ZO-1, β-actin, and EPHA2 proteins are expressed in human SRA 01/04 and mouse αTN4 cells, and occludin protein is expressed in SRA 01/04 cells. Furthermore, they demonstrate that *Occludin* mRNA is expressed in αTN4 cells and *E-cadherin* mRNA is expressed in both SRA 01/04 and αTN4 cells. Expression of the NHS-A isoform of *NHS* in these cell lines has been previously reported by our group [5].

### Protein localization

**Figure 4:**
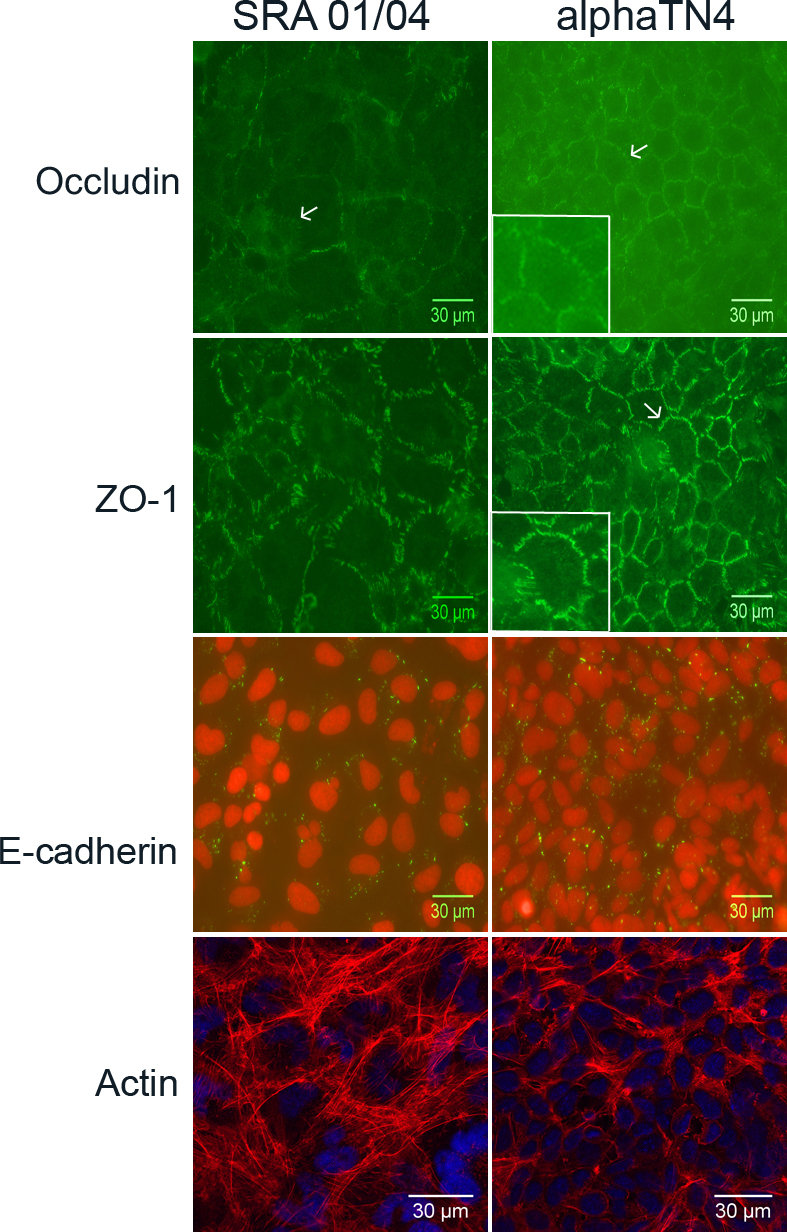
Subcellular localization of cellular junctions associated and cytoskeletal proteins in human SRA 01/04 and mouse αTN4 lens epithelial cells. Endogenous occludin, zona occludin-1 (ZO-1) and E-cadherin proteins were respectively immunolabeled with the anti-occludin, anti-ZO-1, and anti-E-cadherin antibodies. The cytoskeletal actin was stained with phalloidin-TRITC. The labeled protein is indicated on the left. Occludin (green) localized to the cellular periphery as punctate strands perpendicular to the cell boundary (arrow) in both SRA 01/04 and αTN4 cells. In the αTN4 cell panel inset shows magnified view of the cell pointed with an arrow. In some cells, cytoplasmic localization of the protein was also observed. ZO-1 (green) also localized to the cellular periphery in a strand-like pattern perpendicular to the cell boundary in both cell types. In the αTN4 cell panel, arrow points to a cell with strand-like localization; inset shows magnified view of the same cell. Cytoplasmic localization of ZO-1 was also observed in some cells. E-cadherin (green) localized as punctate dots in the cytoplasm in both the cell lines; nuclei (red) stained with propidium iodide. The absence of a signal in each cell type upon hybridization with the secondary antibody alone as a negative control (not shown) proved specificity of localization of each labeled protein. Actin (red) staining showed its localization as radial filaments in the cytoplasm in both the cell types; nuclei (blue) labeled with DAPI. Occludin, ZO-1 and E-cadherin labeling was detected by epifluorescence microscopy using a 40× objective. Actin staining was detected by confocal microscopy using a 60× objective and further digital magnification. Representative images from two independent experiments are shown.

To determine subcellular distribution of the proteins of interest in SRA 01/04 and αTN4 cells, immunolabeling was performed in confluent cultures of these cells. Immunolabeling with the anti-occludin antibody revealed peripheral localization of the protein as punctate strands perpendicular to the cell boundary in both cell types (Figure 4, occludin panels). The strand-like peripheral distribution was more obvious in SRA 01/04 cells due to their larger cell size than in αTN4 cells. In some SRA 01/04 and αTN4 cells cytoplasmic localization of the protein was also observed. Immunolabeling of the ZO-1 protein showed punctate peripheral localization similar to that of occludin in the two cell lines (Figure 4, ZO-1 panels). The ZO-1 signal intensity was stronger and the immunopositive strands at the cellular periphery appeared longer than those observed with the anti-occludin antibody, particularly in SRA 01/04 cells. Some cytoplasmic distribution of the ZO-1 protein was also seen in both SRA 01/04 and αTN4 cells. E-cadherin localizes to the peripheral membrane in polarized epithelial cells [28]. However, immunolabeling of E-cadherin in SRA 01/04 and αTN4 cells showed localization of the protein in the perinuclear region and in a punctate fashion in the cytoplasm (Figure 4, E-cadherin panels). Using the same anti-E-cadherin antibody as used in this study, we previously reported peripheral membrane localization of the E-cadherin protein in MDCK cells [5]. Thus, the intracellular localization of E-cadherin seen in SRA 01/04 and αTN4 cells is specific.

Actin is involved in regulating intercellular junctions and plays an important role in cell adhesion, cell polarity, cell migration, and cell survival [29,30]. In epithelial cells with well defined intercellular contacts, it forms a circumferential ring parallel to the cell membrane [31]. In the present study, in SRA 01/04 and αTN4 cells stained for actin, the protein was present in radially arranged filaments in the cytoplasm (Figure 4, actin panels).

**Figure 5:**
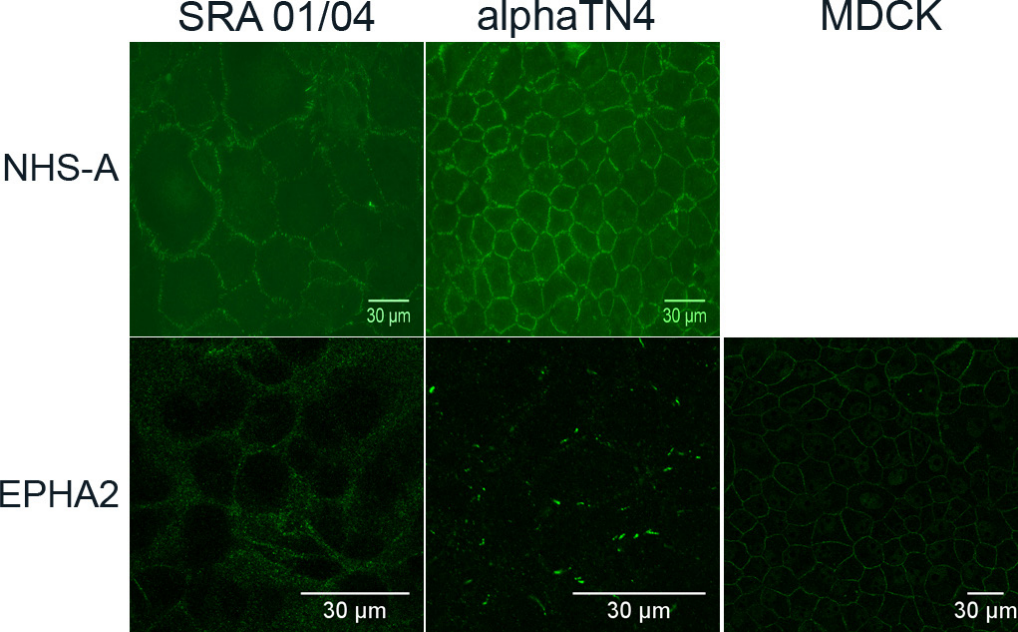
Subcellular localization of NHS-A and EPHA2 proteins in human SRA 01/04 and mouse αTN4 lens epithelial cells. Endogenous NHS-A and EPHA2 proteins were immunolabeled respectively with the anti-NHS and anti-EphA2 antibody. NHS-A localized to the cellular periphery as punctate strands perpendicular to the cell boundary in both the cell types. EPHA2 showed uniformly speckled localization in the cytoplasm in SRA 01/04 cells; in αTN4 cells it localized in the cytoplasm as discrete specks; in positive control Madin-Darby canine kidney (MDCK) cells, it localized to the cellular periphery. The absence of a signal in each cell type upon hybridization with the secondary antibody alone as a negative control (not shown) proved specificity of localization of each labeled protein. NHS-A labeling was detected by epifluorescence microscopy using a 40× objective. EPHA2 labeling was detected by confocal microscopy using a 60× objective and further digital magnification. Representative images from two independent experiments are shown.

Both SRA 01/04 and αTN4 cells express the NHS-A isoform of *NHS*. This isoform interacts with the TJ protein ZO-1 in polarized epithelium in culture and in the mammalian lens [6]. NHS-A is also involved in regulating actin remodeling and cell morphology [15]. Its distribution in SRA 01/04 and αTN4 cells has not been investigated before; we determined this in the present study. Immunolabeling with the anti-NHS antibody revealed localization of the protein at the peripheral cell membrane in both SRA 01/04 and αTN4 cells (Figure 5, NHS-A panels). The protein localized in a strandlike pattern perpendicular to the cell membrane in αTN4 cells. A similar localization pattern but lower signal intensity was observed in SRA 01/04 cells. Weak nuclear localization of the protein was also observed in some cells in both cell lines (data not shown).

EPHA2 is a class A Eph receptor, involved in cell-cell adhesion and repulsion [32]. It localizes to cell-cell contact sites and regulates paracellular permeability and cadherin-based AJs in epithelia [33]. In SRA 01/04 and αTN4 cells, labeled for EPHA2, the protein localized in the cytoplasm (Figure 5, EPHA2 panels). The cytoplasmic localization in SRA 01/04 cells had a speckled appearance and in some cells appeared vesicular, whereas in αTN4 cells it was seen as well defined elongated specks. In MDCK cells used as a positive control, as expected, the protein localized to the cellular periphery at sites of cell-cell contact.

## Discussion

Expression analysis of the TJ proteins occludin and ZO-1 and the AJ protein E-cadherin in SRA 01/04 and αTN4 cells showed that these proteins or their encoding mRNAs are expressed in these lens-derived cell lines. While the ZO-1 protein in both the cell lines, and occludin in SRA 01/04 cells were detectable by western blotting, E-cadherin was not. In addition, occludin was barely detectable in αTN4 cells. However, *Occludin* mRNA in αTN4 cells and *E-cadherin* mRNA in both the cell lines were detected by RT–PCR, albeit after considerable cycles of amplification of the templates (see Material and Methods), thus suggesting lower level expression of these genes in the respective cell lines and supporting the western blotting data. The relatively lower level expression of these genes was also supported by amplification of glyceraldehyde 3-phosphate dehydrogenase (*GAPDH*) mRNA from the same cDNAs after fewer amplification cycles (30 cycles; data not shown). Consistent with lower levels of expression, weak positive immunoreactivity to the occludin protein in αTN4 cells and to the E-cadherin protein in SRA 01/04 and αTN4 cells was observed with antibodies specific to each protein (Figure 4). Furthermore, the presented expression data demonstrate expression of the cytoskeletal actin, and the protein product of the cataract-related gene *EPHA2* in SRA 01/04 and αTN4 cells.

All the proteins investigated in the present study are expressed in the mammalian lens and localize to cell-cell contacts between lens epithelial cells, between lens fiber cells, and/or to the epithelium-fiber interface [5,11,34]. In SRA 01/04 and αTN4 cells, only occludin, ZO-1, and NHS-A proteins localized at the peripheral cell membrane, indicating that these proteins are present at cell-cell contacts (Figure 4 and Figure 5). On the other hand, localization of E-cadherin, actin, and EPHA2 proteins in the cytoplasm in these cells suggests their absence from cell-cell contacts. The localization patterns of NHS-A, ZO-1, and occludin in these cells correlate with those in epithelial cells such as MDCK and Caco-2 [5,6,15]. While in polarized MDCK and Caco-2 cells these proteins form a continuous belt around the cell, in SRA 01/04 and αTN4 cells, they are associated with punctate strands between adjacent cells (Figure 4 and Figure 5). ZO-1 colocalizes and interacts with NHS-A and occludin in polarized MDCK cells [6]. Whether it colocalizes and interacts with these proteins in SRA 01/04 and αTN4 cells will be the subject of a future investigation. Localization of ZO-1 and occludin to strands between adjacent cells is consistent with dependence of occludin on ZO-1 for its recruitment to TJs [35]; localization of NHS-A and ZO-1 to these strands agrees with the correlated localization of these proteins to the cell membrane in polarized MDCK cells [6]. Based on the localization pattern of these three proteins, it seems that SRA 01/04 and αTN4 cells form TJs. Nevertheless, the localization patterns of E-cadherin, actin, and EPHA2 proteins in these cells (Figure 4 and Figure 5) do not correlate with their localization in polarized epithelial cells [7,31,36]. As actin is involved in AJ assembly [18] and E-cadherin-based AJs are regulated by EPHA2 [37], localization of these three proteins at cell-cell contacts is interdependent, which may explain their collective mislocalization in the cytoplasm in SRA 01/04 and αTN4 cells. Furthermore, AJ assembly precedes TJ assembly in epithelial cells because TJ formation is dependent upon AJ formation [31,38]. However, the absence of E-cadherin and EPHA2 from sites of cell-cell contact in SRA 01/04 and αTN4 cells suggests the absence of E-cadherin-based AJs in these cells. Localization of occludin to punctate strands between adjacent cells along with weak expression and cytoplasmic localization of E-cadherin has been reported in CPC-2 human choroid plexus carcinoma cells and attributed to dysregulation of AJs [39]. As SRA 01/04 and αTN4 cells have been transformed with the SV40 large-T antigen, they are in some ways similar to cancerous cells; therefore, it is possible that they have dysregulated AJs and lack E-cadherin based AJs. This may further explain the cytoplasmic localization of EPHA2 in these cells. Similar formation of TJs in the absence of E-cadherin-based AJs and localization of ZO-1 and occludin to TJs has been reported in thyrocytes from E-cadherin conditional knockout mice [40]. In the absence of E-cadherin-based AJs, the SRA 01/04 and αTN4 cells may form N-cadherin based or nestin-afadin-based AJs. Expression of N-cadherin in the mouse lens epithelium [11] supports such a possibility.

TJs in SRA 01/04 and αTN4 cells have a strandlike appearance as opposed to the beltlike appearance in polarized epithelia like MDCK cells [41]. This indicates that the former cells do not form well defined barrier epithelia even in confluent cultures. This idea is consistent with the reported lower transepithelial resistance of αTN4 cells (130±7.3 Ω.cm^2^) compared to that of MDCK cells (613±35 Ω.cm^2^) [42,43]. The transepithelial resistance of SRA 01/04 cells has not been determined. This also correlates with failure of confluent SRA 01/04 and αTN4 cells to arrange in a well defined honeycomb pattern, typically exhibited by polarized MDCK and Caco-2 cells. Taken together, our data suggest the formation of nonpolarized epithelia by SRA 01/04 and αTN4 cells. This may explain why a circumferential actin ring, present in polarized epithelia [44], is absence in these cells.

Interestingly, formation of the circumferential actin ring in Caco-2 cells is dependent upon NHS-A expression as its knockdown leads to disruption of the actin ring, loss of cell shape, and cell spreading [15]. This, however, does not seem to be the case in SRA 01/04 and αTN4 cells as they express NHS-A and yet do not form a circumferential actin ring, thus suggesting that regulation of actin remodeling by NHS-A is cell type dependent, and that NHS-A alone is not sufficient for this regulation.

The localization patterns of the NHS-A and EPHA2 proteins in SRA 01/04 and αTN4 cells indicate that their use as model systems for studying the mechanism of cataractogenesis will depend on the protein under investigation. While these cells may prove useful for investigations into the *NHS* gene, they may not be suitable for studying the *EPHA2* gene.

In conclusion, we demonstrated the expression of the key markers of TJs and AJs and the cytoskeletal protein actin in human SRA 01/04 and mouse αTN4 lens epithelial cells. The localization data suggest the formation of TJs but not E-cadherin-based AJs in these cell lines. Hence, these cells lines likely form nonpolarized epithelia. Investigation of further junctional molecules would reveal the mechanism of cell adhesion in these cell lines.
